# Depressive symptoms in the peripartum: incidence and associated characteristics

**DOI:** 10.1192/j.eurpsy.2022.298

**Published:** 2022-09-01

**Authors:** S. Pompili, A. Mauro, L. Orsolini, V. Salvi, U. Volpe

**Affiliations:** Unit of Clinical Psychiatry, Polytechnic University of Marche, Ancona, Italy, Department Of Neurosciences/dimsc, Ancona, Italy

**Keywords:** EPDS, incidence and characteristic, postpartum depression, peripartum depression

## Abstract

**Introduction:**

The peripartum is a period at high risk for the onset of depressive symptoms. The prevalence of peripartum depression (PD) ranges from 6 to 20% and is burdened with high adverse birth outcomes, poor mother-infant bonding, and a high risk for suicidal ideation and attempts. However, PD is underrecognized and consequently undertreated.

**Objectives:**

We aimed at screening depressive symptoms in women during pregnancy and postpartum, and evaluating the socio-demographic and clinical characteristics associated with depressive symptoms.

**Methods:**

199 women, 55 during pregnancy and 144 in the postpartum period, consecutively admitted to the Perinatal Mental Health Service of Ancona (Italy) were administered a socio-demographic and clinical questionnaire together with the Edinburgh Postnatal Depression Scale (EPDS). Women scoring ≥ 12 at the EPDS were considered screening positive.

**Results:**

Twenty women (10%) were screening-positive. These women were more often foreigners (R2=0,032; β=0,178; p=0,012), single (R2=0,026; β=0,163; p=0,022), with a positive psychiatric family history (R2=0,114; β=-0,337; p=0,001) and more frequently affected by physical comorbidities unrelated to pregnancy (R2=0,03; β=0-0,174; p=0,014). These women also had more gestational comorbidities such as gestational hypertension (R2=0,02; β=-0,154; p=0,030), shortening of uterus neck (R2=0,05; β=-0,234; p=0,001), and miscarriage threats/placental abruption (R2=0,004; β=-0,067; p=0,001).

**Conclusions:**

Our study highlighted the association between depressive symptoms and potentially dangerous gestational comorbidities. Our results further stress the need to screen all women in the peripartum for the presence of depression, in order to identify those at-risk and eventually put in place strategies to prevent further complications to mothers and children.

**Disclosure:**

No significant relationships.

